# Antimicrobial Resistance and Genomic Characterization of Two *mcr-1*-Harboring Foodborne *Salmonella* Isolates Recovered in China, 2016

**DOI:** 10.3389/fmicb.2021.636284

**Published:** 2021-06-15

**Authors:** Yujie Hu, Scott V. Nguyen, Wei Wang, Xin Gan, Yinping Dong, Chang Liu, Xinnan Cui, Jin Xu, Fengqin Li, Séamus Fanning

**Affiliations:** ^1^NHC Key Laboratory of Food Safety Risk Assessment, Food Safety Research Unit (2019RU014) of Chinese Academy of Medical Science, China National Center for Food Safety Risk Assessment, Beijing, China; ^2^UCD-Centre for Food Safety, School of Public Health, Physiotherapy and Population Science, University College Dublin, Dublin, Ireland; ^3^Public Health Laboratory, District of Columbia Department of Forensic Sciences, Washington, DC, United States; ^4^Food Science and Engineering College, Beijing University of Agriculture, Beijing, China; ^5^China Center of Industrial Culture Collection, China National Research Institute of Food and Fermentation Industries, Beijing, China; ^6^Institute for Global Food Security, School of Biological Sciences, Queen’s University Belfast, Belfast, United Kingdom

**Keywords:** *Salmonella*, colistin, antimicrobial resistance (AMR), *mcr-1*, plasmids, China

## Abstract

The *mcr-1* gene mediating mobile colistin resistance in *Escherichia coli* was first reported in China in 2016 followed by reports among different species worldwide, especially in *E. coli* and *Klebsiella*. However, data on its transmission in *Salmonella* are still lacking. This study analyzed the antimicrobial resistance (AMR) profiles and the *mcr-1* gene presence in 755 foodborne *Salmonella* from 26 provinces of mainland, China in 2016. Genomic features of two *mcr-1-*carrying isolates, genome sequencing, serotypes and further resistance profiles were studied. Among the 755 *Salmonella* tested, 72.6% were found to be resistant to at least one antimicrobial agent and 10% were defined as multi-drug resistant (MDR). *Salmonella* Derby CFSA231 and *Salmonella* Typhimurium CFSA629 were *mcr-1*-harboring isolates. Both expressed an MDR phenotype and included a single circular chromosome and one plasmid. Among the 22 AMR genes identified in *S*. Derby CFSA231, only the *mcr-1* gene was localized on the IncX4 type plasmid pCFSA231 while 20 chromosomal AMR genes, including four plasmid-mediated quinolone resistance (PMQR) genes, were mapped within a 64 kb *Salmonella* genomic island (SGI) like region. *S*. Typhimurium CFSA629 possessed 11 resistance genes including an *mcr-1.19* variant and two ESBL genes. Two IS*26*-flanked composite-like transposons were identified. Additionally, 153 and 152 virulence factors were separately identified in these two isolates with secretion system and fimbrial adherence determinants as the dominant virulence classes. Our study extends our concern on *mcr-1*-carrying *Salmonella* in regards to antimicrobial resistance and virulence factors, and highlight the importance of surveillance to mitigate dissemination of *mcr*-encoding genes among foodborne *Salmonella*.

## Introduction

*Salmonella*, one of the top-ranking foodborne pathogens worldwide, is known to cause mild to severe foodborne infections, and has posed a significant public health challenge globally (Jazeela et al., 2020). Antimicrobial compounds are used to treat both human infections and food animal production and evidence suggests that antimicrobial use in food-producing animals contributes to resistance among foodborne *Salmonella* (Crump et al., 2011). This usage also increases the risk of failure when clinical treatment measures are platformed (Bai et al., 2016). On-going surveillance is a necessary step toward monitoring the emergence of multi-drug resistance (MDR) isolates of *Salmonella*.

Colistin (polymyxin E) is considered to be an antimicrobial agent of last-resort for treatment of MDR Gram-negative bacterial infections (Elbediwi et al., 2019). A plasmid-mediated polymyxin resistance mechanism MCR (mobile colistin resistance) and the first *mcr-1* gene was reported in China in 2016 (Liu et al., 2016). The key point of this mechanism is that it encodes a phosphoethanolamine transferase and confers a transferable colistin resistance (Zhao et al., 2017), thus accelerating the therapeutic failure of colistin as a last-resort treatment option for many MDR Gram-negative bacteria (Janssen et al., 2020). Global reports of the identical *mcr-1* gene among several different bacterial species were published shortly thereafter (Sun et al., 2018) along with various publications on related *mcr-1* variants and more divergent *mcr* genes (*mcr-2* ∼ *mcr-10*) (Kluytmans, 2017; Partridge et al., 2018; Osei Sekyere, 2019). According to the proposal for assignment of allele numbers for *mcr* genes and relevant variants (Partridge et al., 2018), *mcr-1.2*7 and *mcr-10* were designated as the latest *mcr-1* variant and *mcr* gene, respectively. Fifteen bacterial genera have been reported to carry *mcr* genes to date, and with the majority within the Enterobacteriaceae (11/15) family. Enterobacteriaceae members *Escherichia coli*, *Salmonella*, *Klebsiella*, and *Aeromonas* of the Aeromonadaceae family are the most common bacteria from which *mcr* genes have been detected with the highest *mcr* gene prevalence reported in non-pathogenic *E. coli* (Elbediwi et al., 2019). China reported the highest number of *mcr*-positive strains in a recent meta-analysis (Elbediwi et al., 2019), and identified *mcr-1* gene in Guangdong, Shanghai, Zhejiang, Hubei, Jiangsu, Sichuan, Shandong, Anhui, Chongqing, Hong Kong, and Taiwan. Based on literature review in public scientific databases, *mcr*-like genes are reported at lower rates in *Salmonella* when compared to *E. coli*, however there have been increased reported numbers of *mcr*-mediated colistin resistance in *Salmonella* spp. from humans, animals, and foods after 2016 (Li et al., 2016; Sun et al., 2018; Lima et al., 2019; Borowiak et al., 2020; Sia et al., 2020).

In this report, a surveillance of the overall antimicrobial resistance (AMR) features of 755 foodborne *Salmonella* isolates in mainland China in 2016 and an investigation of the genomic characteristics of AMR determinants and virulence factors (VFs) of the two *mcr-1-*habouring isolates is conducted to address this data gap.

## Materials and Methods

### Bacterial Isolates

A total of 755 foodborne *Salmonella* isolates were collected from 26 provinces across mainland China in 2016. *Salmonella* isolates were collected from broad food categories including: special nutritional products (powder infant formula, PIF), raw meat and meat products, aquatic and aquatic products, egg and egg products, soy products, frozen drinks, rice and flour products, nuts and seed products, beverages, cocoa products, and local foods among others.

### Antimicrobial Susceptibility Testing (AST) and Screening of the *mcr-1* Gene

All collected *Salmonella* were subjected to AST testing against a panel of compounds (Table 1) by broth micro-dilution using the Biofosun^®^ Gram-negative panel which contained 10 classes (16 kinds) of drugs (Fosun Diagnostics, Shanghai, China). Data obtained was interpreted following recommendations described by Clinical and Laboratory Standards Institute guidelines (CLSI, M100-S28). Additionally, CLSI (M31-A3) and European Committee on Antimicrobial Susceptibility Testing (EUCAST, version 2018) documents were consulted when CLSI M100 standards were not available for some antimicrobial compounds. *E. coli* ATCC^TM^25922 was included as a reference strain.

**TABLE 1:** Antimicrobial susceptibility of 755 foodborne *Salmonella* isolates against to a panel of antimicrobial agents.

**Antimicrobial class**	**Antimicrobial agent (abbreviation)^a^**	**Number of resistant isolates**	**Resistant rate (%)**	**Number of intermediate isolates**	**Intermediate rate (%)**	**Number of susceptible isolates**	**Susceptible rate (%)**
Penicillins	Ampicillin (AMP)	291	38.5	3	0.4	461	61.1
β-Lactam combination agents	Ampicillin/sulbactam (SAM)	263	34.8	32	4.2	460	60.9
Cephalosporins	Cefotaxime (CTX)	89	11.8	4	0.5	662	87.7
	Ceftazidime (CAZ)	45	6.0	11	1.5	699	92.6
	Cephalothin (KF)	103	13.6	47	6.2	605	80.1
	Cefepime (FEP)	40	5.3	7	0.9	708	93.8
Carbapenems	Imipenem (IMP)	0	0.0	0	0.0	755	100.0
	Meropenem (MEM)	0	0.0	0	0.0	755	100.0
Aminoglycosides	Gentamicin (GEN)	83	11.0	0	0.0	672	89.0
Tetracyclines	Tetracycline (TET)	358	47.4	11	1.5	386	51.1
(Fluoro)Quinolones	Nalidixic (NAL)	396	52.5	–	–	359	47.5
	Ciprofloxacin (CIP)	161	21.3	289	38.3	305	40.4
Folate pathway inhibitors	Trimethoprim/sulfamethoxazole (SXT)	179	23.7	–^*e*^	–^*e*^	576	76.3
Phenicols	Chloramphenicol (CHL)	187	24.8	87	11.5	481	63.7
	Florfenicol (FFC)^*b,c*^	170	22.5	65	8.6	520	68.9
Polymyxin	Polymyxin E (Colistin, CT)^*c,d*^	61	8.1	–^*e*^	–^*e*^	694	91.9

*^*a*^Interpretation according to the CLSI guidelines M100-S28, 2018, for all drugs except FFC and CT; ^*b*^interpretation according to the CLSI guidelines M31-A3, 2008; ^*c*^used as a feed additive in animal production; ^*d*^interpretation according to EUCAST clinical breakpoints, 2018; ^*e*^no break-point data.*

All 755 foodborne *Salmonella* isolates were screened for the presence of *mcr-1* gene by qPCR as described previously (Hu et al., 2019). Isolates that carry *mcr-1* gene were selected for further analysis as described below.

### Serotyping and Further AST

Serotypes of *mcr-1* positive isolates were identified by both classical slide agglutination with commercialized antisera (SSI, Denmark) following the Kauffman White Le Minor scheme (WKLM, version 2007, 9th edition), and also by molecular serotyping with xMAP^®^ *Salmonella* Serotyping Assay Kit (SSA, Cat No. AGSSA4502, Luminex, United States) following the manufacturer’s protocol. Extended AST tests using additional selected antimicrobial agents relevant to Enterobacteriaceae, including 13 classes (composing 27 compounds, Table 2), were carried out on the *mcr-1* positive isolates. Extended Spectrum Beta-Lactamase (ESBL) phenotype testing was also performed according to the protocol and breakpoints described in CLSI (M100-S28). *Klebsiella pneumonia*e ATCC^TM^700603 was included as a suitable positive control.

**TABLE 2:** Antimicrobial susceptibility of *Salmonella* isolate CFSA231 and CFSA629 to a further panel of antimicrobial agents and acquired antimicrobial resistance-encoding genes identified in the bacterial genome with online retrieval in Resfinder database.

		**CFSA231**		**CFSA629**		**CFSA231**		**CFSA629**	
**Antimicrobial class**	**Antimicrobial agent (abbreviation)**	**MIC (mg/L)**	**R/I/S^*a*^**	**MIC (mg/L)**	**R/I/S^*a*^**	**Resistance genes**		**Resistance genes**	
						**or point mutation**		**or point mutation**	
						**Chromosome**	**Plasmid**	**Chromosome**	**Plasmid**

β-lactam combination agents	Ampicillin/sulbactam (SAM)	≥32/16	R	≥32/16	R	*bla*_OXA–__1_		*bla*_TEM–__1__B_	*bla*_CTX–M–__14_
Penicillins	Ampicillin (AMP)	≥32	R	≥32	R				
Cephalosporins	Cefotaxime (CTX)	0.12	S	64	R				
	Cefotaxime + clavulanate (CTX + CLA)	0.06/4	–	0.12/4	–				
	Ceftazidime (CAZ)	0.5	S	2	S				
	Ceftazidime + clavulanate (CAZ + CLA)	0.25/4	–	0.25/4	–				
	Cephalothin (KF)	2	S	≥32	R				
	Cefoxitin (FOX)	2	S	8	S				
	Ceftriaxone (CRO)	0.12	S	≥4	R				
	Cefepime (FEP)	2	S	≥16	R				
Carbapenems	Imipenem (IMP)	0.25	S	0.25	S				
	Meropenem (MEM)	0.03	S	0.03	S				
	Ertapenem (ETP)	0.015	S	0.5	S				
Monobactams	Aztreonam (ATM)	0.06	S	4	S				
Aminoglycosides	Gentamicin (GEN)	≥16	R	8	I	*aac(3)-IV, aac(6*′*)-Iaa, aac(6*′*)-Ib-cr, aadA1, aadA2,aaaA2b, aadA8b, aph(3*′*)-Ia, aph(4)-Ia*		*aac(6*′*)-Iaa, aph(3*′′*)-Ib, aph(6)-Id*	*aac(3)-IV, aph(4)-Ia*
	Amikacin(AK)	8	S	1	S				
Tetracyclines	Tetracycline (TET)	≥16	R	≥16	R	*tetA*		*tetB*	
	Tigecycline (TGC)	0.12	S	0.25	S				
(Fluoro)Quinolones	Nalidixic acid (NAL)	≥32	R	≥32	R	ParC T57S; *aac(6*′*)-Ib-cr, oqxA, oqxB, qnrS2*		GyrA D87Y	
	Ciprofloxacin (CIP)	4	R	0.5	I				
Folate pathway inhibitors	Trimethoprim/sulfamethoxazole (SXT)	≥8/152	R	0.25/4.75	S	*sul1, sul2, sul3, dfrA12*		*sul2*	
	Trimethoprim(TMP)	≥16	R	0.25	S				
Phenicols	Chloramphenicol (CHL)	≥32	R	8	S	*catB3, cmlA1, floR*			
	Florfenicol (FFC)^*b,c*^	≥16	R	8	I				
Nitrofurans	Nitrofurantoin (NIT)	32	S	16	S				
Polymyxins	Polymyxin E (Colistin, CT)^*c,d*^	2	S	4	R		*mcr-1.1*		*mcr-1.19*
	Polymyxin B (PB)^*d*^	4	I	4	I				
Fosfomycins	Fosfomycin (FOS)	–^*e*^	–^*e*^	–^*e*^	–^*e*^	*fosA7*			*fosA3*

**R, resistant; I, intermediate; S, susceptible; ^*a*^R/I/S according to the CLSI guidelines M100-S28, 2018; ^*b*^R/I/S according to the CLSI guidelines M31-A3, 2008; ^*c*^used as a feed additive in animal production; ^*d*^R/I/S according to EUCAST clinical breakpoints, 2018; ^*e*^no data*.*

### Plasmid Conjugal Transfer

The transfer ability and frequency of *mcr-*carrying plasmids was investigated by broth mating conjugation experiments with plasmid-free and sodium azide-resistant *E. coli* J53 as the recipient strain. The transconjugants were selected on MacConkey agar plates (Beijing Landbridge, China) supplemented with 100 mg/L sodium azide (Sigma–Aldrich) and 2 mg/L colistin (Sigma–Aldrich). Two different conjugation temperatures (30 and 37°C) were used for the transfer in this study. Transfer frequencies were calculated as the number of transconjugants obtained per recipient. Transfer of *mcr-1* to transconjugants was confirmed by PCR (Liu et al., 2016). The colistin MIC value of the J53 and transconjugants were tested according to the description above.

### DNA Extraction, Whole Genome Sequencing (WGS), Assembly and Annotation

DNA extraction and WGS were carried out for *mcr-1*-carrying isolates to obtain complete genomes. Briefly, a single colony for each isolate was cultured overnight in brain heart infusion (BHI) broth at 37°C. A TIANamp Bacterial DNA extraction kit (DP302, TIANGEN BIOTECH, Beijing, China) was used to extract the genomic DNA from each bacterial culture according to the manufacturer’s instructions, followed by a 10-kbp template library preparation step with PacBio^®^ Template Prep Kit. Sequencing was performed commercially using SMRT^®^ Pacific Biosciences RS II platform (Tianjin Biochip Corporation, Tianjin, China) with C4 sequencing chemistry and P6 polymerase within one SMRT^®^cell.

SMRT^®^ Analysis v2.3.0 was used for demultiplexing, base calling, raw reads quality filtering, and *de novo* assembly according to RS Hierarchical Genome Assembly Process (HGAP) workflow v3.0. Subsequently, Consed software version 28.0 (Gordon and Green, 2013) was used to manually inspect and trim duplicate ends to generate single, complete and closed sequences for each chromosome and plasmid. The genomes assembled from PacBio data were then error corrected by Pilon software (version 1.23) (Walker et al., 2014) with Illumina MiSeq sequencing reads data, of which a library was prepared with a NEBNext^®^ Ultra DNA Library Prep Kit for Illumina (NEB#E7370) followed by sonication fragmentation (350-bp insert), before being loaded on an Illumina HiSeq platform with PE 150 sequencing strategy (Novogene, Beijing, China) with a HiSeq X Ten Reagent Kit v2.5 (Illumina, San Diego, CA, United States). The corrected and assembled contigs were deposited in National Center for Biotechnology Information (NCBI) and automatically annotated using the NCBI Prokaryotic Genomes Automatic Annotation Pipeline (PGAP).

### Genomic Information Mining

Plasmid replicon types (Inc groups) were identified through the center for genomic epidemiology (CGE) website with PlasmidFinder 2.0 (Carattoli et al., 2014). The predicted serotypes were confirmed and the multi-locus sequence typing (MLST) type were identified using *Salmonella In Silico* Typing Resource (SISTR) (Yoshida et al., 2016). CRISPR *loci* in the genomes were predicted using CRISPRfinder (Grissa et al., 2007). Similarly, prophage sequences were identified using the PHAge Search Tool Enhanced Release (PHASTER) (Arndt et al., 2016).

### Assessment of Virulence

Seven extensively used *Salmonella* reference complete genomes LT2 (NC_003197), 14028s (NC_016856), DT104 (NC_022570), CT18 (NC_003198), Ty2 (NC_004631) and two hypervirulent isolates [D23580 (LS997973) and 4/74 (NC_016857)] (Canals et al., 2019), available from GenBank, were used to confirm and compare the presence of *Salmonella* pathogenicity islands (SPIs) and the potential virulence factors (VFs) with two *mcr-1*-carrying isolates in this study by SPIFinder (Roer et al., 2016) and Virulence factor database (VFDB) (Liu et al., 2019). To explore the prevalence of different VFs among various *Salmonella*, a heatmap was made with R and pheatmap, providing for a comparison of the presence or absence of different VFs among the above nine listed genomes and thirteen representative genomes already in the VFDB database with known VFs.

### AMR Analysis

Antimicrobial resistance genes were identified through the Center for Genomic Epidemiology (CGE) website with ResFinder 3.0 (Zankari et al., 2012). DNA sequences of each identified AMR gene regions were selected for detailed BLAST analysis. A MUSCLE alignment was performed in Geneious prime software (version 2019.2.3) between *mcr-1.1* (NG_050417.1) and the two *mcr-1* genes sequences in this study. All genes, plasmids and chromosome sequences used in this study were managed and analyzed by Geneious.

A further comparative sequence alignments were performed in Geneious to identify the nucleotide polymorphism and related amino acid substitution sites compared with the original *mcr-1.1* and all variants which could be found on NCBI to date (*mcr-1.2* ∼ *mcr-1.27*). An unrooted rectangular cladogram tree was also generated for all currently known representatives of the MCR protein subgroups (MCR-1 through MCR-10) and related alleles or variants using the Geneious Tree Builder, with UPGMA tree build method and Jukes-Cator genetic distance model in Geneious software, followed by visualization on EvolView (Subramanian et al., 2019). To better understand the genetic environment of the *mcr-1* locus on plasmids of *mcr-*carrying isolates, these sequences were extracted with Geneious, and compared and displayed using Easyfig v2.2.2 (Sullivan et al., 2011).

### Genome Data Availability

The genome data of chromosome and plasmid sequences of the two *mcr-*1 gene positive *Salmonella* isolates was submitted to the NCBI nucleotide database under BioProject no. PRJNA498334 with Biosample no. of SAMN10291561 and SAMN10291586, and related accession numbers for chromosome and plasmid sequences were: CP033349, CP033350, CP033351, and CP033352.

## Results

### AST for 755 Foodborne *Salmonella* Isolates

**FIGURE 1:**
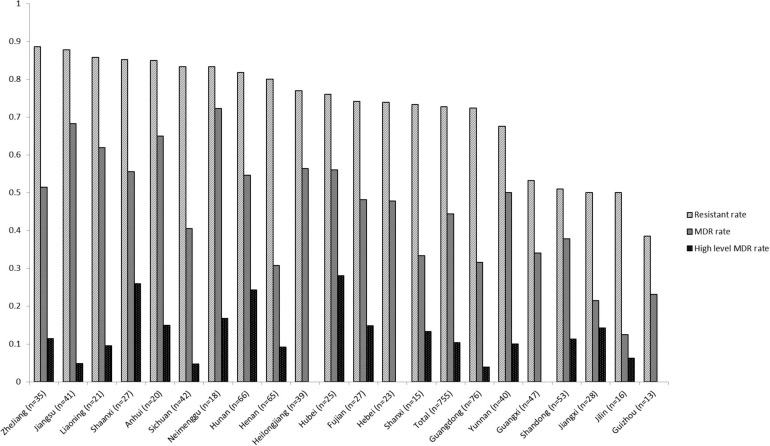
Antimicrobial resistance of foodborne *Salmonella* isolates recovered from different provinces in mainland China, 2016. [Multi-drug resistant (MDR), resistant to three or more classes of antimicrobial agents].

The percentages related to AMR for all 755 *Salmonella* isolates recovered from various foods are shown in Table 1. Among these, 206 isolates (27.3%) were susceptible to all antimicrobial agents and 549 (72.7%) exhibited resistance to at least one compound. The resistance rates were classified into three categories: (1) higher than 34%: NAL, TET, AMP, and SAM; (2) between 11.0 and 24.8%: CHL, SXT, FFC, CIP, KF, CTX and GEN; (3) lower than 10%: CT, CAZ, FEP, IMP, and MEM. Resistance to four cephalosporin-type compounds demonstrated a decreasing trend across the generations of this drug class (KF > CTX/CAZ > FEP). No isolate was resistant to carbapenem-type compounds (IMP and MEM). Sixty five (65/755, 8.6%) isolates were co-resistant to both cefotaxime and ciprofloxacin, two first-line antimicrobial agents used to treat human salmonellosis clinically. One hundred and twenty eight (17.0%), 86 (11.4%), 90 (11.9%), 79 (10.5%), 39 (5.2%), 49 (6.5%), 52 (6.9%), and 26 (3.4%) isolates were resistant to 1, 2, 3, 4, 5, 6, 7, and 8 classes of antimicrobial agents tested, respectively. In total, 134 different AMR profiles were recorded among 549 AMR *Salmonella* isolates. There were 335 isolates (44.4%) were classified as MDR (resistant to three or more classes of antimicrobial agents) and 78 isolates (10.3%) were identified as high level MDR (resistant to seven or eight classes of antimicrobials). Three isolates were co-resistant to 13 different antimicrobial (GEN-AMP-SAM-FEP-SXT-NAL-CHL-TET-CTX-FFC-CAZ-KF-CIP). Nineteen out of 26 provinces returned a resistant rate of higher than 50%, and 9 were recorded to exceed 80% with the highest of 88.6% (31/35, Zhejiang province). Ten of 26 had an MDR rate of no less than 50%, while 4 of them were found to exceed 60% (Inner Mongolia, Jiangsu, Anhui, and Liaoning). The level of MDR rates of Hubei, Shaanxi, and Hunan provinces were higher than 24% (Figure 1). The AMR data of 755 isolates were available in Supplementary data sheet 1.

### Serotyping, Further AST and Plasmid Conjugation for *mcr-1* Gene Positive Isolates

The complete collection was tested for the presence of the *mcr-1* gene by qPCR and two *Salmonella* isolates, CFSA231 and CFSA629, were positive with an *mcr-1* gene detection rate of 0.26% (2/755) among *Salmonella* from mainland China, 2016. The two isolates were recovered from a pork dumpling sample (Huangshi, Hubei) and from an egg sample (Zhongshan, Guangdong), respectively. Based on both serotyping methods mentioned above, CFSA231 and CFSA629 were separately identified as Derby and Typhimurium serotype with the antigen formulas of 1,4,[5],12:f,g:- and 1,4,[5],12:i:1,2. Both isolates demonstrated an MDR phenotype to six or seven classes of antimicrobials but with some notable differences (Table 2). For instance, *S*. Derby CFSA231 expressed resistance to gentamicin, ciprofloxacin, folate pathway inhibitors and phenicols, while resistance against cephalosporins and colistin were observed for *S.* Typhimurium CFSA629. Based on the MIC value change for cefotaxime and ceftazidime in combination with clavulanate compared with when tested alone (Table 2), *S.* Typhimurium CFSA629 was confirmed as an ESBL positive strain whilst *S*. Derby CFSA231 was negative. *mcr*-harboring plasmids of both isolates could transfer into *E. coli* J53 at frequencies as below: pCFSA231, 1.5 × 10^–7^ (30°C) and 2.6 × 10^–6^ (37°C); pCFSA629, 2.1 × 10^–4^–2.0 × 10^–2^ (30°C), and 3.9 × 10^–6^ (37°C).

### Genome Sequence Features, SPIs, Virulence Factors (VFs) and AMR Genes

Both genomes of *S*. Derby CFSA231 and *S*. Typhimurium CFSA629 consisted of a single circular chromosome and a circular plasmid. Details of the genomic features of the bacterial chromosomes and plasmids are shown in Table 3. CRISPR features and prophage information is available in Supplementary Tables 1, 2, respectively. Different serotypes showed different SPI genotypes, and SPI details are summarized in Table 4, all six *S*. Typhimurium strains contain the same SPI genotype independent of STs; Meanwhile, when compared with two *S*. Typhi (CT18 and Ty2), a small SPI type difference was noted and related to the presence of SPI-6, whilst these could be distinguished from other serotypes in having SPI-7 through SPI-14 and C63PI.

**TABLE 3:** Genome sequence features for CFSA231 and CFSA629.

**Strain or plasmid name**	**Serotype, MLST type or plasmid replicon type**	**Number of reads**	**Mean read length (bp)**	**Coverage**	**Size (bp)**	**G + C content**	**Number of coding genes, pseudo genes and RNA genes**
CFSA231	Derby ST40	65,092	8,680	68.13x	4,834,516	52.1%	4,519; 134; 119
pCFSA231	IncX4				33,309	41.9%	
CFSA629	Typhimurium ST34	54,855	9,083	57.39x	4,999,270	52.1%	4,937; 117; 125
pCFSA629	IncHI2A/IncHI2				210,674	45.2%	

**TABLE 4:** Distribution of *Salmonella* pathogenicity island (SPIs) in seven representative genomes of *Salmonella* isolates (Identity threshold: 95%, minimum length: 60%).

***Salmonella* strain**	**CFSA231**	**CFSA629**	**LT2**	**14028S**	**DT104**	**D23580**	**4/74**	**CT18**	**Ty2**
Serotype	Derby	Typhimurium	Typhimurium	Typhimurium	Typhimurium	Typhimurium	Typhimurium	Typhi	Typhi
MLST type	ST40	ST34	ST19	ST19	ST19	ST313	ST19	ST2	ST1
Accession number	CP033350.2	CP033352.2	NC_003197.2	NC_016856.1	NC_022570.1	LS997973.1	NC_016857.1	NC_003198.1	NC_004631.1
SPI-1	−	+	+	+	+	+	+	+	+
SPI-2	+	+	+	+	+	+	+	+	+
SPI-3	+	+	+	+	+	+	+	+	+
SPI-4	+	+	+	+	+	+	+	+	+
SPI-5	−	+	+	+	+	+	+	+	+
SPI-6	−	−	−	−	−	−	−	+	−
SPI-7	−	−	−	−	−	−	−	+	+
SPI-8	−	−	−	−	−	−	−	+	+
SPI-9	−	−	−	−	−	−	−	+	+
SPI-10	−	−	−	−	−	−	−	+	+
SPI-11	−	−	−	−	−	−	−	−	−
SPI-12	−	−	−	−	−	−	−	+	+
SPI-13	−	+	+	+	+	+	+	−	−
SPI-14	−	+	+	+	+	+	+	−	−
C63PI	+	+	+	+	+	+	+	−	−

**FIGURE 2:**
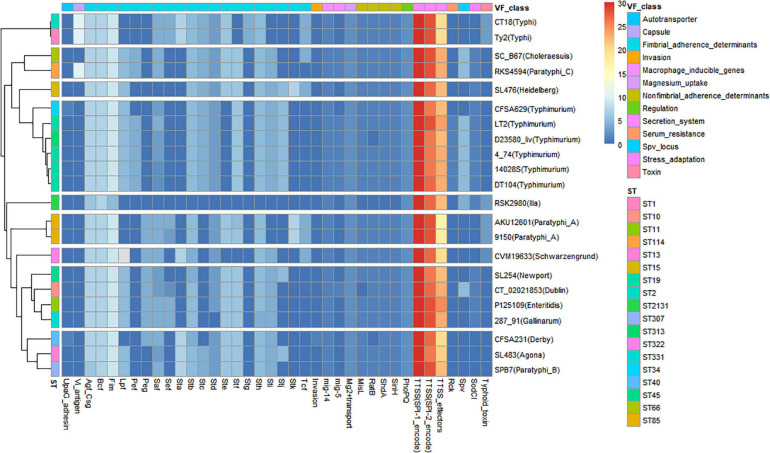
Heatmap showing the relationship of 22 genomes/isolates including *Salmonella* Derby CFSA231 and *Salmonella* Typhimurium CFSA629 and 20 reference genomes based on their differential virulence factor classes. Numbers of virulence factors were represented by different colors, high number were marked with red squares (pixels) and low numbers were represented with blue squares.

A VF heatmap was generated using R for CFSA231, CFSA629 and 20 additional *Salmonella* reference genomes with known VFs (Figure 2). It showed that the bacterial secretion system was the dominant VF class, and the top three VFs included the SPI-1 encoded Type Three Secretion System (T3SS), the SPI-2 encoded T3SS and T3SS effectors denoted by the red and yellow colors in the heatmap (Figure 2). The VF pattern of one *Salmonella* isolate appeared to be more closely related to isolates of the same serotype, rather than to the isolates of any other serotypes, such as *S*. Typhi, *S*. Typhimurium, and *S*. Paratyphi A. CFSA629 ST34 was distinguished from those of *S.* Typhimurium ST19 and ST313, with the main difference being the absence of the following VFs: Pef, Mig-5 and Spv. Six typhoidal *Salmonella*, including two *S*. Typhi and four *S*. Paratyphi were clustered into four distinct clades. As listed in Supplementary Table 3, the VF number of the 22 *Salmonella* genomes varied from 110 (*S*. *enterica* subsp. *arizonae* ser.62:z4,z23:– str. RSK2980) to 176 (*S*. Paratyphi C str. RKS4594). The annotated genes belonging to secretion system and fimbrial adherence virulence classes were the top two VFs for CFSA231 and CFSA629.

**FIGURE 3:**
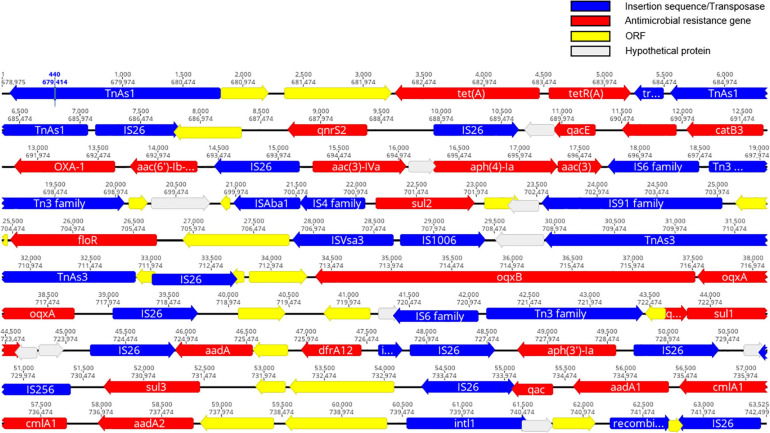
A schematic illustration showing the structural of ∼64kb MDR gene cluster region on chromosome of CFSA231 (created using Geneious software). Antimicrobial resistance (AMR)-encoding genes are indicated in red boxes/arrows. Blue boxes/arrows denote transposon- and integron-associated genes. The individual open reading frame (ORF) are indicated with yellow boxes/arrows. The light gray boxes indicate hypothetical proteins.

The resistance genotypes of CFSA231 and CFSA629 identified by Resfinder were shown in Table 2. In *S*. Derby CFSA231 the *mcr-1* gene was located on an IncX4-type plasmid pCFSA231 along with a *pap2-*encoding gene distal to this site. We identified a number of similar well conserved plasmids differing by less than 4 nucleotides by BLAST on NCBI, and all of which were distributed in *Enterobacteriaceae*, with a high number of these (>80%) being associated with *E. coli*. Except *mcr-1*, all other 21 AMR-encoding genes identified in the genome of CFSA231, including one ESBL gene (*bla*_OXA–__1_), were localized to the bacterial chromosome; one point mutation in the *parC* gene, resulting in an amino acid substitution (T57S) was identified. Four ciprofloxacin resistance-encoding genes [*aac(6′)-Ib-cr*, *oqxA*, *oqxB*, *qnrS2*], that are more commonly associated with plasmid-mediated quinolone resistance (PMQR), were mapped on the chromosome. These 21 genes were located within a 64-kbp locus that contained 83 CDS identified which encoded 23 transposases, 20 chromosomal antimicrobial resistant genes mediating resistance to nine drugs in this study, one class 1 integron gene, one recombinase gene and other genes encoding functional and hypothetical proteins (Figure 3). A query coverage value of 100% and identity of 100% were recorded with a single nucleotide difference identified between this locus and a similar arrangement reported earlier in a chicken-derived *Salmonella* isolate CD-SL01 recovered from chicken in China, 2015-2016 (NCBI accession number: CP028900.1). Both chromosomal genomes were compared using Geneious and the MAFFT Alignment programme with default settings, and were found to have an identity of 99.947% and 2,565 base/residue differences in total and CD-SL01 was devoid of any plasmids.

In *S*. Typhimurium CFSA629, a mutation in *gyrA* giving rise to a D87Y substitution was found; six and five antimicrobial resistant genes were located on the chromosome and plasmid, respectively and two different ESBL genes (*bla*_TEM–__1__B_ and *bla*_CTX–M–__14_) mapped to them each. The *mcr-1* gene was also located on plasmid, an IncHI2 type plasmid pCFSA629, which possessed a single copy of the IS*Apl1* element along with a gene encoding the PAP2 family protein flanking the left and the right side of the *mcr-1* gene. There was two IS*26* composite-like transposonal modules in this isolate which was rich in insertion sequences and transposons: one consisting of IS*26*-*fosA3*-IS*1182*-*bla*_CTX–M–__4_-IS*26* and another one was IS*26*-[*aac(3)-IV]*-[*aph(4)-Ia]*-IS*6* family-Tn*3*-IS*26*.

### Comparison and Relationship Between *mcr* Genotypes and MCR Variants

**FIGURE 4:**
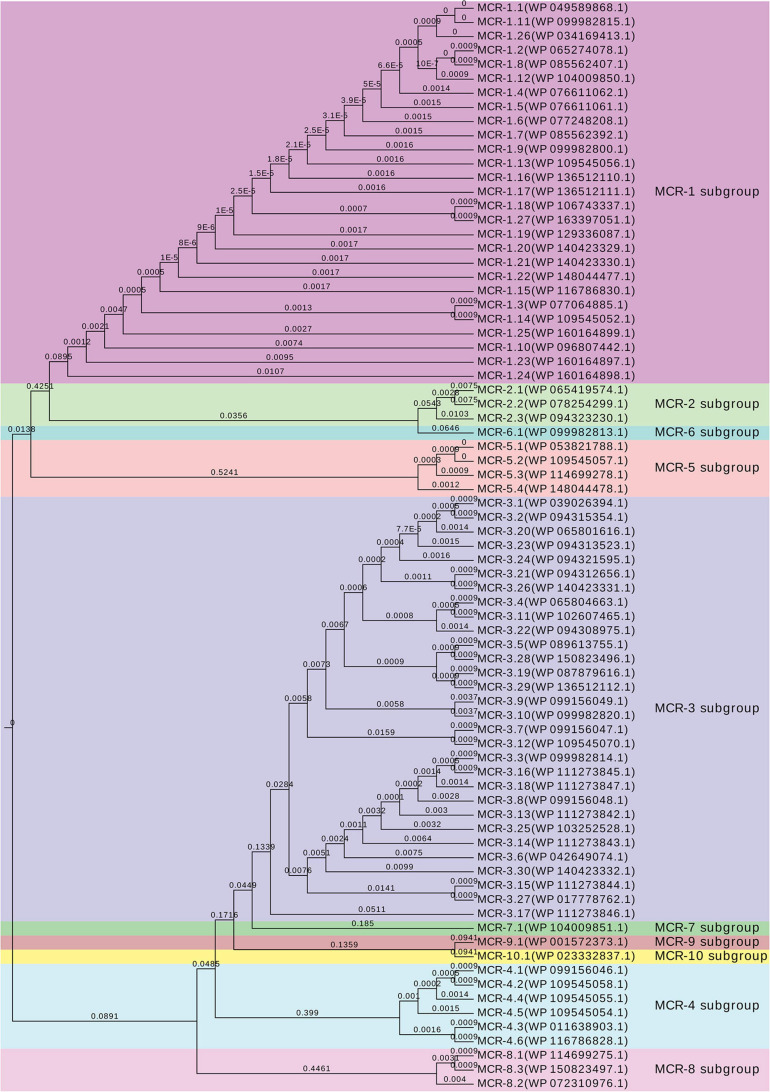
Genetic relationships between MCR protein subgroups and related alleles. UPGMA rectangular cladogram tree of the deduced amino acid sequences of putative phosphoethanolamine transferase belonged to different subgroups was constructed using Geneious prime v2019.0.4 software with Jukes–Cantor genetic distance model, and visualized by EvolView online tool (v2.0, https://www.evolgenius.info/evolview/). NCBI accession numbers are listed following by the MCR alleles and available in GenBank.

An *mcr-1* variant was identified and confirmed on pCFSA629 by Geneious (Hu et al., 2021), and was assigned with allele number *mcr-1.19* (MK490674.1, G1,534A) and corresponding protein MCR-1 allele number of MCR-1.19 (QBC35984.1, Val512-to-Ile) in GenBank. Compared with the *mcr-1.1*, no base mutations were observed in the *mcr-1* gene on pCFSA231. The comparative alignment between 26 *mcr-1* variants and *mcr-1.1*, as well as the amino acid differences among their related *mcr-1* coding protein variants was shown in Table 5. One single nucleotide difference was observed among most *mcr-1* genotypes (*n* = 21), and a dinucleotide difference existed in three of these variants. In terms of MCR-1.1 with 541 amino acids, a trinucleotide duplication was found in *mcr-1.11*, resulting in an additional amino acid for an MCR-1 variant of 542 amino acids, while *mcr-1.15* and *mcr-1.26* with 540 amino acids, arising from a single nucleotide polymorphism (SNP) located at initiation codon. It should be noted that three *mcr-1* variants (*mcr-1.10*, *mcr-1.23*, and *mcr-1.24*) had more than 12 nucleotide differences relative to *mcr-1.1*, leading to at least 7 amino acid differences from MCR-1.1. The non-rooted rectangular cladogram showed two distinguished clusters among ten MCR protein sub-groups (Figure 4). In the case of the genetic distances within the MCR-1 subgroup, MCR-1.10, MCR-1.23, and MCR-1.24, which exhibited the most amino acid differences relative to MCR-1.1 were also the furthest in genetic distance from MCR-1.1, followed by MCR-1.25, MCR-1.14, MCR-1.3, and MCR-1.15.

**TABLE 5:** Nucleotide/amino acid changes of *mcr-1*/MCR-1 alleles/variants compared with *mcr-1.1*/MCR-1.1 (the accession numbers emanated from Reference gene browser with gene family “*mcr-1*,” database version: 2020–05-04.1. Nucleotide and amino acid differences were identified by Geneious software).

**Allele/variant**	**RefSeq protein**	**Refseq nucleotide**	**GenBank protein**	**GenBank nucleotide**	**Nucleotide differences compared with *mcr-1.1***	**Amino acid differences compared with MCR-1.1**	**First discovered host bacteria**
*mcr-1.1*	WP_049589868.1	NG_050417.1	AKF16168.1	KP347127.1	N/A	N/A	*Escherichia coli*
*mcr-1.2*	WP_065274078.1	NG_051170.1	OBY14952.1	LXQO01000025.1	A8T	Gln3Leu	*Klebsiella pneumoniae*
*mcr-1.3*	WP_077064885.1	NG_052861.1	ANJ15621.1	KU934208.1	AA111-112GG	Ile38Val	*Escherichia coli*
*mcr-1.4*	WP_076611062.1	NG_052664.1	APM87143.1	KY041856.1	G1318A	Asp440Asn	*Escherichia coli*
*mcr-1.5*	WP_076611061.1	NG_052663.1	APM84488.1	KY283125.1	C1354T	His452Tyr	*Escherichia coli*
*mcr-1.6*	WP_077248208.1	NG_052893.1	AQK48217.1	KY352406.1	G1263A, G1607A	Arg536His	*Salmonella enterica*
*mcr-1.7*	WP_085562392.1	NG_054678.1	AQQ11622.1	KY488488.1	G643A	Ala215Thr	*Escherichia coli*
*mcr-1.8*	WP_085562407.1	NG_054697.1	AQY61516.1	KY683842.1	A8G	Gln3Arg	*Escherichia coli*
*mcr-1.9^*a*^*	WP_099982800.1	NG_055582.1	ASK38392.2	KY964067.1	T1238C	Val413Ala	*Escherichia coli*
*mcr-1.10^*b*^*	WP_096807442.1	NG_055583.1	ASK49940.1	MF176238.1	–	–	*Moraxella* sp.
*mcr-1.11^*c*^*	WP_099982815.1	NG_055784.2	ATM29809.1	KY853650.2	GTG19-21dup	Val7dup	*Escherichia coli*
*mcr-1.12*	WP_104009850.1	NG_056412.1	BBB21811.1	LC337668.1	G9C	Gln3His	*Escherichia coli*
*mcr-1.13*	WP_109545056.1	NG_057466.1	AVM85874.1	MG384739.1	G465A	Met155Ile	*Escherichia coli*
*mcr-1.14*	WP_109545052.1	NG_057460.1	ARA74236.1	KX443408.2	AA111-112GG, G591A	Ile38Val, Met197Ile	*Klebsiella pneumoniae*
*mcr-1.15^*d*^*	WP_116786830.1	NG_061610.1	AXL06756.1	MG763897.1	AT1-2TA, C836A	Met1del, Thr279Lys	*Klebsiella pneumoniae*
*mcr-1.16*	WP_136512110.1	NG_064787.1	QBG64271.1	MK568462.1	C952A	Arg318Ser	*Escherichia coli*
*mcr-1.17*	WP_136512111.1	NG_064788.1	QBG64272.1	MK568463.1	G410C	Ser137Thr	*Escherichia coli*
*mcr-1.18*	WP_106743337.1	NG_064789.1	–	PGLM01000025.1	T25G	Tyr9Asp	*Escherichia coli*
*mcr-1.19*	WP_129336087.1	NG_065449.1	QBC35984.1	MK490674.1	G1534A	Val512Ile	*Salmonella enterica*
*mcr-1.20*	WP_140423329.1	NG_065450.1	SPQ84451.1	LS398440.1	A184C	Met62Leu	*Escherichia coli*
*mcr-1.21*	WP_140423330.1	NG_065451.1	QCU55424.1	MK965883.1	C1234T	Pro412Ser	*Escherichia coli*
*mcr-1.22*	WP_148044477.1	NG_065944.1	QDO71694.1	MN017134.1	C1277T	Ser426Phe	*Escherichia coli*
*mcr-1.23^*e*^*	WP_160164897.1	NG_067235.1	QHD57408.1	MN873697.1	–	–	*Salmonella enterica*
*mcr-1.24^*f*^*	WP_160164898.1	NG_067236.1	QHD64700.1	MN879257.1	–	–	*Escherichia coli*
*mcr-1.25*	WP_160164899.1	NG_067237.1	QHD64702.1	MN879259.1	G41C, C565G	Ser14Thr, Leu189Val	*Escherichia coli*
*mcr-1.26^*d*^*	WP_034169413.1	NG_068217.1	NEU93872.1	JAAGSA010000042.1	T2C	Met1del	*Escherichia coli*
*mcr-1.27*	WP_163397051.1	NG_068218.1	NEU89143.1	JAAGSB010000042.1	A26G	Tyr9Cys	*Escherichia coli*

**^*a*^Using start codon that matches other mcr-1 genes rather than the one in the original INSDC entry. ^*b*^Details not listed for 39 nucleotide differences between mcr-1.10 and mcr-1.1 as well as 7 amino acid differences between MCR-1.10 and MCR-1.1. ^*c*^Dup, duplication of nucleotides/amino acids at positions indicated. ^*d*^Del, deletion of amino acids at positions indicated. In comparison of mcr-1.1, the nucleotide at position 1 and 2 (AT) of mcr-1.15 mutate into TA, and also the nucleotide at position 2 (T) of mcr-1.26 mutate into C, resulting in a unavailable translation initiation codon of ATG at 1-3 and a retroposed codon from 4-6(ATG). ^*e*^Details not listed for 12 nucleotide differences between mcr-1.23 and mcr-1.1 as well as 9 amino acid differences between MCR-1.23 and MCR-1.1. ^*f*^Details not listed for 13 nucleotide differences between mcr-1.24 and mcr-1.1 as well as 10 amino acid differences between MCR-1.24 and MCR-1.1.**

### Genetic Environment Context of *mcr-1* Gene in pCFSA231 and pCFSA629

**FIGURE 5:**
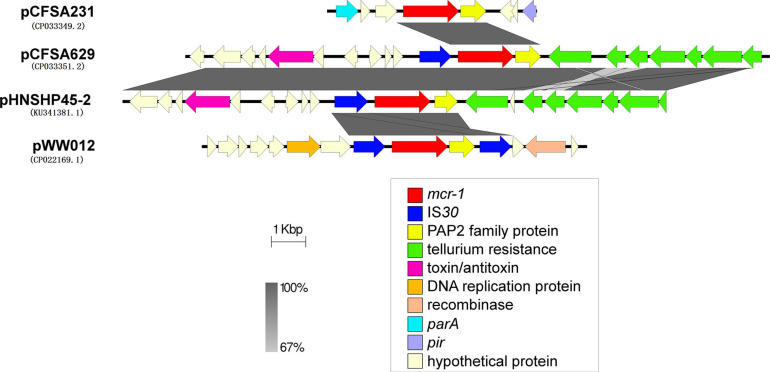
Genetic environments related to *mcr-1* gene in bacterial plasmids. The figure was generated by Easyfig (v2.2.2). Plasmids marked with “pCFSA” were carried by two *mcr-1* positive *Salmonella* isolates in this research, and plasmid pWW012 belonged to a previous research in our lab (accession number: CP022169), while pHNSHP45-2 (accession number: KU341381) belonged to *Escherichia coli* strain SHP45, which is the first isolate reported harboring *mcr-1* gene. Confirmed and putative open reading frames (ORFs) are indicated by block arrows and their orientations with different colors, and arrow size is proportional to the predicted ORF length. *mcr-1* gene is indicated by a red arrow, while genes encoding mobile elements (insertion sequence, IS) are indicated by blue arrows. Regions of homology between the plasmids ranging from 67 to 100% are indicated by the graded shaded regions between sequences.

The *mcr-1* locus of plasmid pCFSA231 and pCFSA629 were extracted and compared with the relevant *mcr-1* locus on plasmids pHNSHP45-2 (KU341381.1) from *E. coli* and pWW012 (CP022169.1) from *Salmonella* (Figure 5). It showed that the gene structures near *mcr-1* on plasmids varied but shared the same regions (genes encoding MCR-1 and PAP2 family proteins), with different presence of insertion sequences (ISs). pCFSA231 had no IS and pCFSA629 obtained only one IS*Apl1;* a tellurium resistance gene cluster was located downstream PAP2 coding gene of pCFSA629 and pHNSHP45-2; pWW012, the *mcr-1*-carrying plasmid from our previous study, consisted of an IS-*mcr-1*-PAP2-IS module which is an IS*Apl1*-flanked composite transposon (Tn*6330*).

## Discussion

*Salmonella* are important foodborne zoonotic pathogens often linked to cases of gastroenteritis and bacteremia, and are one of the leading causes of global bacterial food poisoning worldwide (Threlfall, 2002). The antimicrobial resistance expressed by *Salmonella* is spreading in both developed and developing countries (Parisi et al., 2018). In this study, similar antimicrobial resistance for foodborne *Salmonella* isolates was observed with previous study in China (Hu et al., 2017). The AMR rate recorded against ciprofloxacin (21.3%) indicated a comparatively statistically significant increase when compared with the previous year (Hu et al., 2017). A potential explanation for the high ciprofloxacin intermediate rate in this study (38.3%) may be due to the updated and extended range of criteria in the CLSI guidelines, but this finding partly signal a continuously reduced susceptibility trend among foodborne *Salmonella* to ciprofloxacin in recent years. Gong et al. (2019) found that almost 90% of *S*. Indiana were resistant to both ciprofloxacin and cefotaxime and 8.6% of the tested isolates in this study demonstrated this feature of resistance, posing a serious threat to public health and ongoing surveillance is clearly necessary in regard to monitoring the emergence of resistance and identify transmission routes.

In this study different serotypes of *Salmonella* possessed different SPI and VF genotypes, and the impact of these differences for a comparison of serotype on virulence potential remains unclear. For example, the hypervirulent invasive non-typhoidal (iNTS) *S.* Typhimurium ST313 str. D23580 could not easily be distinguished from other *S.* Typhimurium of different ST types based on the VFs, since there are several mechanisms that could contribute to its pathogenicity in iNTS (Carden et al., 2015, 2017; Hammarlöf et al., 2018). Even within a serovar, differences in SPI content may also affect virulence potential such as the presence or absence of the *avrA* secreted effector gene in certain *S.* Montevideo lineages (Nguyen et al., 2018). In comparison to many studies of resistance prediction from AMR genes, increased emphasis or the joint on functional transcriptomics, proteomics and genomics techniques are necessary for virulence potential investigation/prediction and pathogenicity modeling.

In this study, two *mcr-1*-carrying foodborne *Salmonella* isolates were recovered among 755 strains (0.26%). According to our latest data related to more than 3,800 foodborne *Salmonella* recovered in mainland China between 2011 and 2019 (data not published), 14 *mcr-1*-harboring isolates were detected corresponding to a positivity rate of 0.4%, which could be regarded as a low prevalence rate. Similar results were reported previously for isolates cultured from clinical, food or food-producing animals (Carnevali et al., 2016; Cui et al., 2017; Lu et al., 2019; Luo et al., 2020). Susceptibility testing of CFSA231 and CFSA629 against colistin, recorded MIC values of 2 mg/L, are consistent with *mcr-1* mediated low-level colistin resistance (2–8 mg/L) (Zhang et al., 2019). Acquisition of this gene could also facilitate further selection of chromosomal mutants in some cases, leading to high-level colistin resistance (HLCR) (Zhang et al., 2019). Thus screening and surveillance for the *mcr-1* in bacteria of importance to human health is critical. Besides their distinct geographic and sample origins, the two *mcr-1*-positive *Salmonella* isolates in this study exhibited different MDR profiles. All 27 compounds tested in the extended AST tests in this study are listed in CLSI and EUCAST and are used in human and veterinary settings. An integrated *One Health* based surveillance system is crucial in tracking these developments, and it should be a more initiative monitoring model which could focus on the antimicrobial resistance dissemination among human beings, animals and environments at the same time (Luo et al., 2020).

The AMR determinants detected in this report were in coherence with the AMR patterns obtained by AST. In the case of *S*. Derby CFSA231 all resistance genes, except for *mcr-1*, were mapped to the chromosome, including four PMQR genes that are more commonly associated with plasmid. The 64 kbp putative SGI-like MDR region in this isolate was largely similar to the *Salmonella* Genomic Island 1 (SGI1) with an ACSSuT phenotype reported earlier (Mulvey et al., 2006), differing in the numbers of mobile genetic elements (MGEs) it contained. BLAST analysis of this locus identified genetically homologous regions in both chromosomal- and plasmid-based sequences in different species including *Salmonella*, *E. coli*, and *K. pneumoniae*, suggesting that this putative SGI1-like region might have already disseminated among the Enterobacteriaceae family, and this will cause a potential of a processed *copy-out-paste-in* transpositional event resulting in dissemination and stabilization of these related resistant genes. The corresponding resistance genes that zoonotic *Salmonella* have acquired are more commonly located on plasmids, in transposons, gene cassettes, or variants of the SGI1 and SGI2 loci (Michael and Schwarz, 2016), thus studies exploring the dissemination of this putative SGI1-like locus may provide further insights into its evolution (Michael and Schwarz, 2016).

The *mcr-1* gene has been found in plasmids with different Inc types such as IncI2, IncHI1, IncHI2, IncP, IncX4, IncFI, and IncFIB (Zhi et al., 2016; Poirel et al., 2017). Based on the epidemiological study (Lu et al., 2019), the *mcr-1*-carrying IncHI2 type plasmid was originally identified in *Salmonella* isolated from diarrhoeal outpatients in Shanghai in 2014 and increasingly detected after the summer of 2015, representing the primary replicon type in 2016. It was reported that foods had played important roles in the expansion of *mcr-1*-carrying IncHI2 plasmids among different members of the Enterobacteriaceae family before 2016, and what is of increasing concern, with usage of antimicrobials other than polymyxins, co-selection of *mcr-1* may happen due to the MDR phenotype and conjugative ability of the IncHI2 plasmids (Zhi et al., 2016). For instance, a *Salmonella* IncHI2 plasmid that predate the era of mass antibiotic usage has been sequenced with no detectable AMR genes (Nguyen et al., 2017), but modern IncHI2 plasmids encoding multiple AMR genes are predominant in MDR *Salmonella* (Chen et al., 2016). Thus, there is a potential for capture of *mcr-1* in MDR *Salmonella* due to co-selection by other antimicrobials other than polymyxin. The prevalence of the *mcr* gene is higher in resistant bacterial isolates cultured from food-producing animals when compared to those cultured from humans. This may be due to the application of antimicrobials in agricultural for production purposes. Although use of colistin in agriculture production has been recently banned in China and Brazil, surveillance must be maintained as increasing clinical usage of polymyxins may also contribute to the risk of dissemination of *mcr* markers in nosocomial settings (Osei Sekyere, 2019).

The co-existence of plasmid-mediated *mcr-1* and carbapenemase-encoding genes has been identified in Enterobacteriaceae and there are increasing reports of this co-occurrence worldwide. Isolates of both clinical and animal origin combining *bla-*encoding genes (such as *bla*_NDM–__1_, *bla*_NDM–__5_, *bla*_NDM–__9_, *bla*_OXA–__48_, *bla*_KPC–__2_, and *bla*_VIM–__1_) with *mcr-1* gene may signal the risk of the emerging of strains expressing pan-drug resistance (PDR) (Yang et al., 2016; Lai et al., 2017; Wang et al., 2018; Le-Vo et al., 2019). It is important to note that the presence of *mcr-1* gene on the chromosome in recent studies (Falgenhauer et al., 2016; Zurfluh et al., 2016; Yamaguchi et al., 2020) suggest that the *mcr-1* gene could become more stable through vertical inheritance in *mcr-1*-carrying isolates.

The role of MGEs such as transposons has made an important contribution to the AMR rapid dissemination by horizontal gene transfer under selective pressure imposed by the antimicrobial usage (Snesrud et al., 2018). *mcr-1* gene could be found in various combinations with one or two copies of IS*Apl1* or devoid of the IS element with different replicon types (Li et al., 2017). Snesrud et al. (2018) presented representative sequences of the four general *mcr-1* structures identified to date: (a) composite transposon Tn*6330* that is thought to mediate the initial mobilization of event; (b) a single-ended structure with a distal copy of IS*Apl1*; (c) a structure lacking both copies of IS*Apl1*; (d) and a single-ended structure with a proximal copy of IS*Apl1* only. We can imagine a genetic element with the loss of IS*Apl1* may provide a more stable *mcr-1* state, especially when integrated in the chromosome. In this study, pCFSA231 and pCFSA629 are classified in the structures described in (c) and (b) above, respectively. They did not contain two ISs and the likelihood of losing or moving *mcr-1* gene relatively decreased, however, our results still suggest that plasmids of various replicon types may contribute to the *mcr-1* gene movement and global spread, accelerating the frequency of colistin resistance worldwide.

## Conclusion

Colistin is an important antibacterial agent used for treating MDR Gram-negative bacteria infections. Intrinsic colistin resistance located on the chromosome was generally thought to be non-transferable until the detection of MCR, a transferable polymyxin resistance reported globally among various bacterial species with comparatively few descriptions in *Salmonella.* In the context of the rapid spreading trend of the clinical *mcr-1*-harboring *Salmonella* and continuous discoveries of novel *mcr* genes and related variants, we report on the AMR profiles of a set of foodborne *Salmonella* cultured from mainland China and describe the complete genomes of two *mcr-1*-positive *Salmonella* isolates, including a *S.* Typhimurium isolate with an *mcr-1-*variant. Improved surveillance is important for understanding the dissemination of *mcr* genes among foodborne *Salmonella* around the world.

## Data Availability Statement

The genome data of chromosome and plasmid sequences of the two *mcr-1* gene positive *Salmonella* isolates were submitted to the NCBI nucleotide database under BioProject numbers PRJNA498334 with Biosample No. of SAMN10291561 and SAMN10291586, and related accession numbers for chromosome and plasmid sequences were: CP033349, CP033350, CP033351, and CP033352.

## Author Contributions

YH performed the literature search. YH, FL, and SF designed the research. YH, SN, WW, XG, YD, CL, and XC performed the experiments and collected the data. YH, SN, WW, and JX analyzed and interpreted the data and finished the figures and tables. YH and SN wrote the manuscript. FL and SF reviewed and edited the manuscript. All authors read and approved the manuscript. YH, SN, FL, and SF have accessed and verified the underlying data.

## Conflict of Interest

The authors declare that the research was conducted in the absence of any commercial or financial relationships that could be construed as a potential conflict of interest.
