# Supplementary material for: Antimicrobial Resistance and Genomic Characterization of Two mcr-1-Harboring Foodborne Salmonella Isolates Recovered in China, 2016
Source: Front Microbiol. 2021 Jun 15;12:636284. doi: 10.3389/fmicb.2021.636284 (PMC8239406; doi:10.3389/fmicb.2021.636284)
Supplement: Supplementary file 2 [file Table_1.docx]

**TABLE S1** CRISPR loci of *Salmonella* Derby CFSA231 and *S.* Typhimurium CFSA629.

| CRISPRs candidates | **CRISPR ID** | **Isolates** | DR consensus sequence | **Number of spacers** | **CRISPR length (bp)** | **DR length (bp)** | **Spacer length (bp)** |
| --- | --- | --- | --- | --- | --- | --- | --- |
| Confirmed CRISPRs | CP033352.2_Crispr_2 | CFSA629 | CGGTTTATCCCCGCTGGCGCGGGGAACAC | 9 | 578 | 29 | 32 |
|  | CP033352.2_Crispr_3 | CFSA629 | CGGTTTATCCCCGCTGGCGCGGGGAACAC | 21 | 1311 | 29 | 32, 33 |
|  | CP033352.2_Crispr_4 | CFSA629 | CGGTTTATCCCCGCTGGCGCGGGGAACAC | 24 | 1493 | 29 | 32, 33 |
|  | CP033350.2_Crispr_1 | CFSA231 | GTGTTCCCCGCGCCAGCGGGGATAAACCG | 13 | 821 | 29 | 32 |
|  | CP033350.2_Crispr_2 | CFSA231 | GTGTTCCCCGCGCCAGCGGGGATAAACCG | 28 | 1737 | 29 | 32, 33 |
| Questionable CRISPRs | CP033352.2_PossibleCrispr_1 | CFSA629 | GCTTCAGTGGCGAACGTCGTGAA | 1 | 100 | 23 | 55 |
