# Supplementary material for: Antimicrobial Resistance and Genomic Characterization of Two mcr-1-Harboring Foodborne Salmonella Isolates Recovered in China, 2016
Source: Front Microbiol. 2021 Jun 15;12:636284. doi: 10.3389/fmicb.2021.636284 (PMC8239406; doi:10.3389/fmicb.2021.636284)
Supplement: Supplementary file 3 [file Table_2.docx]

**TABLE S2** prophage identified in the genomes of *S.* Derby CFSA231 and *S.* Typhimurium CFSA629.

| **Isolates** | **Region** | **Region Length** | **Complete^a^** | **Score** | **Total Proteins** | Region Position | **Most Common Phage** | **GC %** |
| --- | --- | --- | --- | --- | --- | --- | --- | --- |
| CFSA231 | 1 | 3.9Kb | incomplete | 20 | 6 | [3364263-3368235 info_outline](http://phaster.ca/submissions/ZZ_aae766fafb#region_dna0) | PHAGE_Entero_UAB_Phi20_NC_031019(2) | 41.81% |
|  | 2 | 61.7Kb | intact | 150 | 56 | [3602277-3664048 info_outline](http://phaster.ca/submissions/ZZ_aae766fafb#region_dna1) | PHAGE_Entero_SfV_NC_003444(41) | 50.77% |
|  | 3 | 20.4Kb | questionable | 70 | 24 | [4460539-4480961 info_outline](http://phaster.ca/submissions/ZZ_aae766fafb#region_dna2) | PHAGE_Burkho_BcepMu_NC_005882(14) | 50.60% |
| CFSA629 | 1 | 34.7Kb | intact | 120 | 38 | [666101-700890 info_outline](http://phaster.ca/submissions/ZZ_785b5a9be5#region_dna0) | PHAGE_Haemop_HP2_NC_003315(17) | 50.12% |
|  | 2 | 43.2Kb | intact | 150 | 59 | [1541917-1585180 info_outline](http://phaster.ca/submissions/ZZ_785b5a9be5#region_dna1) | PHAGE_Salmon_SE1_NC_011802(22) | 47.40% |
|  | 3 | 6.3Kb | incomplete | 20 | 9 | [1832222-1838584 info_outline](http://phaster.ca/submissions/ZZ_785b5a9be5#region_dna2) | PHAGE_Shigel_SfIV_NC_022749(2) | 45.84% |
|  | 4 | 7Kb | incomplete | 40 | 6 | [2196817-2203852 info_outline](http://phaster.ca/submissions/ZZ_785b5a9be5#region_dna3) | PHAGE_Cronob_vB_CsaM_GAP32_NC_019401(2) | 52.23% |
|  | 5 | 40.6Kb | intact | 150 | 52 | [2273913-2314532 info_outline](http://phaster.ca/submissions/ZZ_785b5a9be5#region_dna4) | PHAGE_Phage_Gifsy_2_NC_010393(34) | 49.89% |
|  | 6 | 11.5Kb | incomplete | 50 | 8 | [3119367-3130925 info_outline](http://phaster.ca/submissions/ZZ_785b5a9be5#region_dna5) | PHAGE_Entero_HK630_NC_019723(2) | 43.39% |
|  | 7 | 24.1Kb | incomplete | 60 | 13 | [3118767-3142884 info_outline](http://phaster.ca/submissions/ZZ_785b5a9be5#region_dna6) | PHAGE_Salmon_Fels_1_NC_010391(3) | 46.49% |
|  | 8 | 55.2Kb | intact | 120 | 67 | [3246937-3302141 info_outline](http://phaster.ca/submissions/ZZ_785b5a9be5#region_dna7) | PHAGE_Salmon_118970_sal3_NC_031940(92) | 50.09% |
|  | 9 | 15.3Kb | incomplete | 50 | 17 | [3535464-3550830 info_outline](http://phaster.ca/submissions/ZZ_785b5a9be5#region_dna8) | PHAGE_Salmon_118970_sal3_NC_031940(5) | 49.80% |
|  | 10 | 62.6Kb | intact | 150 | 60 | [3922026-3984694 info_outline](http://phaster.ca/submissions/ZZ_785b5a9be5#region_dna9) | PHAGE_Phage_Gifsy_1_NC_010392(51) | 51.34% |
|  | 11 | 7.8Kb | incomplete | 30 | 8 | [4250203-4258040 info_outline](http://phaster.ca/submissions/ZZ_785b5a9be5#region_dna10) | PHAGE_Salmon_SJ46_NC_031129(2) | 51.70% |
|  | 12 | 20.4Kb | questionable | 70 | 23 | [4606322-4626741 info_outline](http://phaster.ca/submissions/ZZ_785b5a9be5#region_dna11) | PHAGE_Burkho_BcepMu_NC_005882(14) | 50.58% |

^a^ Intact (score > 90), Questionable (score 70-90), Incomplete (score < 70)
