# Supplementary material for: Antimicrobial Resistance and Genomic Characterization of Two mcr-1-Harboring Foodborne Salmonella Isolates Recovered in China, 2016
Source: Front Microbiol. 2021 Jun 15;12:636284. doi: 10.3389/fmicb.2021.636284 (PMC8239406; doi:10.3389/fmicb.2021.636284)
Supplement: Supplementary file 4 [file Table_3.docx]

**TABLE S3** Protential virulence factors identified in *S.* Derby CFSA231 and *S.* Typhimurium CFSA629 and other 20 reference genomes identified by VFDB database.

| **VF classes** | **number** | **CFSA231** | **CFSA629** | **LT2** | **14028S** | **DT104** | **CT18** | **Ty2** | **D23580_liv** | **4_74** | **SL483** | **P125109** | **RSK2980** | **SC_B67** | **CT_02021853** | **287_91** | **SL476** | **SL254** | **AKU12601** | **9150** | **SPB7** | **RKS4594** | **CVM19633** |
| --- | --- | --- | --- | --- | --- | --- | --- | --- | --- | --- | --- | --- | --- | --- | --- | --- | --- | --- | --- | --- | --- | --- | --- |
|  | **ST** | **40** | **34** | **19** | **19** | **19** | **2** | **1** | **313** | **19** | **13** | **11** | **2131** | **66** | **10** | **331** | **15** | **45** | **85** | **85** | **307** | **114** | **322** |
|  | **Serotype** | **Derby** | **Typhimurium** | **Typhimurium** | **Typhimurium** | **Typhimurium** | **Typhi** | **Typhi** | **Typhimurium** | **Typhimurium** | **Agona** | **Enteritidis** | **IIIa 62:z4,z23:--** | **Choleraesuis** | **Dublin** | **Gallinarum** | **Heidelberg** | **Newport** | **Paratyphi A** | **Paratyphi A** | **Paratyphi B** | **Paratyphi C** | **Schwarzengrund** |
| **Accession No.** | |  |  |  |  |  |  |  |  |  |  |  |  |  |  |  |  |  |  |  |  |  |  |
| Autotransporter | UpaG adhesin | 1 | 1 | 0 | 0 | 0 | 0 | 0 | 0 | 0 | 0 | 0 | 0 | 0 | 0 | 0 | 0 | 0 | 0 | 0 | 0 | 0 | 0 |
| Capsule | Vi antigen | 0 | 0 | 0 | 0 | 0 | 10 | 10 | 0 | 0 | 0 | 0 | 0 | 0 | 0 | 0 | 0 | 0 | 0 | 0 | 0 | 10 | 0 |
| Fimbrial adherence determinants | Agf_Csg | 7 | 7 | 7 | 7 | 7 | 7 | 7 | 7 | 7 | 7 | 7 | 6 | 7 | 7 | 7 | 7 | 7 | 7 | 7 | 6 | 7 | 7 |
|  | Bcf | 7 | 7 | 7 | 7 | 7 | 7 | 7 | 7 | 7 | 7 | 7 | 7 | 7 | 7 | 7 | 7 | 7 | 7 | 7 | 7 | 7 | 7 |
|  | Fim | 9 | 9 | 9 | 9 | 9 | 9 | 9 | 9 | 9 | 9 | 9 | 5 | 9 | 9 | 9 | 9 | 9 | 9 | 9 | 9 | 9 | 9 |
|  | Lpf | 0 | 5 | 5 | 5 | 5 | 0 | 0 | 5 | 5 | 5 | 5 | 0 | 5 | 5 | 5 | 5 | 5 | 0 | 0 | 5 | 5 |  |
|  | Pef | 0 | 0 | 4 | 4 | 4 | 0 | 0 | 4 | 4 | 0 | 0 | 0 | 4 | 0 | 0 | 0 | 0 | 0 | 0 | 0 | 4 | 0 |
|  | Peg | 4 | 0 | 0 | 0 | 0 | 0 | 0 | 0 | 0 | 0 | 4 | 0 | 0 | 4 | 4 | 0 | 4 | 4 | 4 | 0 | 0 | 4 |
|  | Saf | 4 | 3 | 4 | 2 | 2 | 4 | 4 | 3 | 3 | 3 | 4 | 0 | 4 | 3 | 4 | 0 | 3 | 4 | 4 | 4 | 4 | 4 |
|  | Sef | 0 | 0 | 0 | 0 | 0 | 4 | 4 | 0 | 0 | 0 | 4 | 0 | 0 | 2 | 4 | 0 | 0 | 4 | 3 | 0 | 0 | 0 |
|  | Sta | 7 | 0 | 0 | 0 | 0 | 7 | 7 | 0 | 0 | 7 | 0 | 0 | 0 | 0 | 0 | 0 | 0 | 0 | 0 | 7 | 0 | 7 |
|  | Stb | 4 | 5 | 5 | 5 | 5 | 5 | 5 | 5 | 5 | 5 | 5 | 0 | 5 | 5 | 5 | 5 | 4 | 5 | 5 | 5 | 5 | 4 |
|  | Stc | 0 | 4 | 4 | 4 | 4 | 4 | 4 | 4 | 4 | 4 | 0 | 0 | 4 | 0 | 0 | 4 | 0 | 0 | 0 | 4 | 4 | 0 |
|  | Std | 3 | 3 | 3 | 3 | 3 | 3 | 3 | 3 | 3 | 3 | 3 | 0 | 3 | 3 | 0 | 3 | 3 | 3 | 3 | 3 | 3 | 3 |
|  | Ste | 6 | 0 | 0 | 0 | 0 | 6 | 6 | 0 | 0 | 6 | 6 | 0 | 6 | 6 | 6 | 6 | 6 | 6 | 6 | 6 | 6 | 0 |
|  | Stf | 6 | 6 | 6 | 6 | 6 | 0 | 0 | 6 | 6 | 6 | 6 | 0 | 6 | 6 | 6 | 6 | 6 | 5 | 6 | 6 | 6 | 0 |
|  | Stg | 0 | 0 | 0 | 0 | 0 | 4 | 4 | 0 | 0 | 0 | 0 | 0 | 0 | 0 | 0 | 0 | 0 | 0 | 0 | 0 | 0 | 0 |
|  | Sth | 5 | 5 | 5 | 5 | 5 | 5 | 5 | 5 | 5 | 5 | 5 | 0 | 5 | 5 | 4 | 5 | 5 | 5 | 5 | 5 | 5 | 5 |
|  | Sti | 4 | 4 | 4 | 4 | 4 | 0 | 0 | 4 | 4 | 4 | 4 | 0 | 4 | 4 | 4 | 4 | 4 | 0 | 0 | 4 | 4 | 4 |
|  | Stj | 0 | 5 | 5 | 5 | 5 | 0 | 0 | 5 | 5 | 5 | 0 | 0 | 0 | 0 | 0 | 5 | 5 | 0 | 0 | 0 | 0 | 0 |
|  | Stk | 0 | 0 | 0 | 0 | 0 | 0 | 0 | 0 | 0 | 0 | 0 | 0 | 0 | 0 | 0 | 7 | 0 | 7 | 7 | 0 | 0 | 0 |
|  | Tcf | 0 | 0 | 0 | 0 | 0 | 4 | 4 | 0 | 0 | 0 | 0 | 0 | 4 | 0 | 0 | 4 | 0 | 4 | 4 | 0 | 0 | 4 |
| Invasion | Invasion | 1 | 0 | 0 | 0 | 0 | 0 | 0 | 0 | 0 | 0 | 0 | 0 | 0 | 0 | 0 | 0 | 0 | 0 | 0 | 0 | 0 | 0 |
| Macrophage inducible genes | mig-14 | 1 | 1 | 1 | 1 | 1 | 1 | 1 | 1 | 1 | 1 | 1 | 1 | 1 | 1 | 1 | 1 | 1 | 1 | 1 | 1 | 1 | 1 |
|  | mig-5 | 0 | 0 | 1 | 1 | 1 | 0 | 0 | 1 | 1 | 0 | 0 | 0 | 1 | 1 | 0 | 0 | 0 | 0 | 0 | 0 | 1 | 0 |
| Magnesium uptake | Mg2+ transport | 2 | 2 | 2 | 2 | 2 | 2 | 2 | 2 | 2 | 2 | 2 | 2 | 2 | 2 | 2 | 2 | 2 | 2 | 2 | 2 | 2 | 2 |
| Nonfimbrial adherence determinants | MisL | 1 | 1 | 1 | 1 | 1 | 1 | 1 | 1 | 1 | 1 | 1 | 0 | 1 | 0 | 1 | 1 | 1 | 1 | 1 | 1 | 1 | 1 |
|  | RatB | 0 | 1 | 1 | 1 | 1 | 1 | 1 | 0 | 1 | 1 | 1 | 0 | 1 | 1 | 1 | 1 | 1 | 1 | 1 | 1 | 1 | 1 |
|  | ShdA | 1 | 1 | 1 | 1 | 1 | 1 | 1 | 1 | 1 | 1 | 1 | 0 | 1 | 1 | 1 | 1 | 1 | 1 | 1 | 1 | 1 | 1 |
|  | SinH | 1 | 1 | 1 | 1 | 1 | 1 | 1 | 1 | 1 | 1 | 1 | 0 | 1 | 1 | 1 | 1 | 1 | 1 | 1 | 1 | 1 | 1 |
| Regulation | PhoPQ | 2 | 2 | 2 | 2 | 2 | 2 | 2 | 2 | 2 | 2 | 2 | 2 | 2 | 2 | 2 | 2 | 2 | 2 | 2 | 2 | 2 | 2 |
| Secretion system | TTSS(SPI-1 encode) | 30 | 30 | 30 | 30 | 30 | 30 | 30 | 30 | 30 | 30 | 30 | 30 | 30 | 30 | 30 | 30 | 30 | 30 | 30 | 30 | 30 | 30 |
|  | TTSS(SPI-2 encode) | 27 | 26 | 28 | 26 | 27 | 28 | 28 | 26 | 26 | 26 | 28 | 27 | 28 | 27 | 28 | 27 | 26 | 28 | 28 | 28 | 28 | 27 |
|  | TTSS_effectors | 20 | 22 | 24 | 23 | 23 | 20 | 20 | 22 | 23 | 21 | 25 | 22 | 23 | 23 | 23 | 22 | 22 | 17 | 18 | 22 | 23 | 20 |
| Serum resistance | Rck | 0 | 0 | 1 | 1 | 1 | 0 | 0 | 1 | 1 | 0 | 0 | 0 | 0 | 0 | 0 | 0 | 0 | 0 | 0 | 0 | 0 | 0 |
| Spv locus | Spv | 0 | 0 | 5 | 5 | 5 | 0 | 0 | 5 | 5 | 0 | 0 | 4 | 5 | 5 | 0 | 0 | 0 | 0 | 0 | 0 | 5 | 0 |
| Stress adaptation | SodCI | 0 | 1 | 1 | 1 | 1 | 0 | 0 | 1 | 1 | 0 | 1 | 1 | 1 | 1 | 1 | 1 | 1 | 0 | 0 | 0 | 1 | 0 |
| Toxin | Typhoid toxin | 0 | 0 | 0 | 0 | 0 | 3 | 3 | 0 | 0 | 0 | 0 | 3 | 0 | 0 | 0 | 0 | 0 | 3 | 3 | 0 | 0 | 2 |
| Total |  | 152 | 151 | 167 | 162 | 163 | 169 | 169 | 161 | 163 | 162 | 162 | 110 | 170 | 161 | 156 | 166 | 156 | 157 | 158 | 160 | 176 | 146 |
